# Identification of Bax-interacting proteins in oligodendrocyte progenitors during glutamate excitotoxicity and perinatal hypoxia–ischemia

**DOI:** 10.1042/AN20130027

**Published:** 2013-12-23

**Authors:** Sopio Simonishvili, Mohit Raja Jain, Hong Li, Steven W. Levison, Teresa L. Wood

**Affiliations:** *Department of Neurology & Neuroscience, New Jersey Medical School Cancer Center, Rutgers Biomedical & Health Sciences, Newark, NJ 07101, U.S.A.; †Center for Advanced Proteomic Research and Department of Biochemistry and Molecular Biology, New Jersey Medical School Cancer Center, Rutgers Biomedical & Health Sciences, Newark, NJ 07101, U.S.A.

**Keywords:** apoptosis, Bcl-xL, Bid, cofilin, insulin-like growth factor 1 (IGF-I), oligodendrocyte, ACN, acetonitrile, ADF, actin depolymerizing factor, AF488, Alexa Fluor 488, AF546, Alexa Fluor 546, CCA, common carotid artery, CL, contralateral, CNS, central nervous system, DMEM, Dulbecco’s modified Eagle’s medium, FBS, fetal bovine serum, FGF-2, fibroblast growth factor-2, H–I, hypoxia–ischemia, IGF, insulin-like growth factor, IL, ipsilateral, IP, immunoprecipitation, MEM, minimal essential media, OPC, oligodendrocyte progenitor cell, PIC, protease inhibitor cocktail, tBid, truncated Bid, VDAC, voltage-dependent anion channel

## Abstract

OPC (oligodendrocyte progenitor cell) death contributes significantly to the pathology and functional deficits following hypoxic-ischemic injury in the immature brain and to deficits resulting from demyelinating diseases, trauma and degenerative disorders in the adult CNS. Glutamate toxicity is a major cause of oligodendroglial death in diverse CNS disorders, and previous studies have demonstrated that AMPA/kainate receptors require the pro-apoptotic protein Bax in OPCs undergoing apoptosis. The goal of the present study was to define the pro-apoptotic and anti-apoptotic effectors that regulate Bax in healthy OPCs and after exposure to excess glutamate *in vitro* and following H–I (hypoxia–ischemia) in the immature rat brain. We show that Bax associates with a truncated form of Bid, a BH3-only domain protein, subsequent to glutamate treatment. Furthermore, glutamate exposure reduces Bax association with the anti-apoptotic Bcl family member, Bcl-xL. Cell fractionation studies demonstrated that both Bax and Bid translocate from the cytoplasm to mitochondria during the early stages of cell death consistent with a role for Bid as an activator, whereas Bcl-xL, which normally complexes with both Bax and Bid, disassociates from these complexes when OPCs are exposed to excess glutamate. Bax remained unactivated in the presence of insulin-like growth factor-1, and the Bcl-xL complexes were protected. Our data similarly demonstrate loss of Bcl-xL–Bax association in white matter following H–I and implicate active Bad in Bax-mediated OPC death. To identify other Bax-binding partners, we used proteomics and identified cofilin as a Bax-associated protein in OPCs. Cofilin and Bax associated in healthy OPCs, whereas the Bax–cofilin association was disrupted during glutamate-induced OPC apoptosis.

## INTRODUCTION

Oligodendrocytes are myelin-producing cells of the CNS (central nervous system) essential for the health and proper function of neural circuitry. Alterations in oligodendrocyte numbers and in myelination are associated with a number of neurological and psychiatric disorders that include multiple sclerosis, spinal cord injury, adult stroke, schizophrenia and several neurodegenerative diseases (Almad et al., 2011; Lee et al., 2012; Matute and Ransom, 2012; Bankston et al., 2013; Philips et al., 2013). It has been established that there is a maturation-dependent vulnerability of oligodendroglia to injury (Oka et al., 1993; Back et al., 2001; McTigue and Tripathi, 2008; Bradl and Lassmann, 2010) with the immature progenitors significantly more vulnerable than mature oligodendrocytes. For example, using a neonatal rat model of hypoxic-ischemic brain injury, Back and colleagues found that the white matter injury involved apoptotic death of the late OPCs (oligodendrocyte progenitor cells) (Back et al., 2001, 2002). Late OPCs also were found to be vulnerable to cell death in other models of neonatal white matter injury (Vottier et al., 2011; Falahati et al., 2013). Oligodendrocyte progenitors are especially vulnerable to increased levels of glutamate, which has been implicated in white matter damage after neonatal H–I (hypoxia–ischemia), spinal cord injury and multiple sclerosis (Petito et al., 1998; Ness et al., 2001; Park et al., 2004). Axons, and rather surprisingly, oligodendroglia themselves, are major source of extracellular glutamate in white matter (Back et al., 2007). Oligodendroglia express AMPA/kainate receptors (Patneau et al., 1994) which mediate the excitotoxic death of the OPCs in developing white matter (Yoshioka et al., 1995, 1996; Matute et al., 1997; McDonald et al., 1998a, 1998b; Yoshioka et al., 2000; Alberdi et al., 2002; Ness and Wood, 2002; Sanchez-Gomez et al., 2003). Whereas oligodendroglia also express NMDA receptors, NMDA receptor expression appears to be most prominent in mature stages of the lineage (Karadottir et al., 2005; Salter and Fern, 2005; Micu et al., 2006).

Several studies have begun to define the death pathway initiated in OPCs by high levels of extracellular glutamate. In a prior study, we demonstrated that excess glutamate activates AMPA/kainate receptors and Ca^2+^ influx in late OPCs resulting in translocation of the pro-apoptotic protein Bax to the mitochondria, cytochrome *c* release, activation of caspase 9 and caspase 3, nuclear fragmentation and cell death (Ness et al., 2004). Moreover, we showed that IGF (insulin-like growth factor)-I sustains Akt phosphorylation in OPCs and prevents Bax mitochondrial translocation and apoptosis in excitotoxic conditions. Subsequently, Pang and colleagues demonstrated that IGF-I also protects OPCs from TNFα (tumor necrosis factor α) cytotoxicity in a dose-dependent manner by preventing Bax translocation from the cytosol to mitochondria via activation of PI3K (phosphoinositide 3-kinase)/Akt (Pang et al., 2007). Deletion of Bax in mice attenuated oligodendrocyte death after spinal cord injury *in vivo* (Dong et al., 2003) and after staurosporine, cyclosporine A or AMPA/kainite-mediated insults *in vitro* (Dong et al., 2003; Sanchez-Gomez et al., 2011). Moreover, blocking Bax translocation to mitochondria significantly protected rat and mouse oligodendrocytes from AMPA- and kainate-induced damage (Sanchez-Gomez et al., 2011).

The mechanisms by which Bax is regulated in healthy cells and activated during apoptosis appears varied depending on cell type and type of toxicity. Both direct activation and indirect Bax activation in apoptosis have been described (Labi et al., 2006; Hacker and Weber, 2007; Willis et al., 2007; Chipuk and Green, 2008; Giam et al., 2008). In the direct activation model, an ‘activator’ BH3-only domain protein is required to directly activate Bax or its related multidomain pro-apoptotic protein, Bak. In healthy cells the pro-survival Bcl-family members Bcl2 or Bcl-xL sequester the BH3 protein. In the indirect model of Bax activation, BH3 proteins activate apoptosis by binding and neutralizing the pro-survival proteins, allowing Bax/Bak to homo-oligomerize and permeabilize the mitochondria.

In previous studies we determined that glutamate-mediated Bax translocation and apoptosis, as well as IGF-I protection from these events in OPCs, was downstream of calcium entry following activation of AMPA/kainite receptors and independent of changes in Bcl-2, Bcl-xL, or Bax protein levels (Ness et al., 2004). The goal of the present study was to determine Bax suppressors in healthy OPCs and Bax activators in glutamate-induced excitotoxicity *in vitro* and in white matter following H–I in the immature brain.

## MATERIALS AND METHODS

MEM (minimal essential media), DMEM (Dulbecco's modified Eagle's medium)/F-12, FBS (fetal bovine serum) and trypsin were purchased from Gibco/Invitrogen, and papain from Worthington. Cell culture supplements, and L-glutamic acid monosodium salt hydrate were purchased from Sigma Chemical Compan. Recombinant rat IGF-I was purchased from Upstate Biochemicals. Recombinant human FGF-2 (fibroblast growth factor-2) was purchased from R&D Systems. Rabbit polyclonal antibodies against Bax (N20), Bcl-xL, and Bid were obtained from Santa Cruz Biotechnology, against Bax6A7 from BD Transduction Labs, and against Bad and p-Bad(Ser^155^) from Cell Signaling Technology. Antibodies against β-actin were from Sigma–Aldrich; cleaved caspase 3 and VDAC (voltage-dependent anion channel) antibodies were from Cell Signaling Technology. Cofilin antibodies were from Santa Cruz Biotechnology and Cell Signaling Technology. Chemical reagents Chaps and NP-40 (Igepal) were from Sigma–Aldrich. Common laboratory chemicals were purchased from either Sigma or VWR International.

### Cell culture and treatment conditions

All experiments were performed in accordance with research guidelines set forth by the Society for Neuroscience Policy on the use of animals in neuroscience research. Animal protocols were reviewed and approved by the IACUC committee of NJMS/UMDNJ. Mixed glia were isolated from newborn Sprague–Dawley rat forebrain cortices as previously described (McCarthy and de Vellis, 1980; Ness and Wood, 2002; Ness et al., 2004). Briefly, tissues were enzymatically digested with trypsin and DNase I and then mechanically dissociated and plated in MEM containing 10% FBS and 30% glucose with antibiotics. The mixed glial cells were grown in T75 flasks until they were confluent (10 days). Microglia were separated from the cultures by shaking the flasks on a rotary shaker for 1.5 h at 260 rev./min. OPCs were isolated following an additional 18 h. OPCs were seeded on to poly-D-lysine-coated T75 flasks at density of 2×10^4^ /cm^2^ in N2S, composed of 66% N2B2 media [DMEM/F12, supplemented with 0.66 mg/ml BSA, 10 ng/ml D-Biotin, 20 nM progesterone, 100 μM putrecsine, 50 μg/ml apo-transferrin, 100 units/ml penicillin/streptomycin, 5 μg/ml insulin and 34% B104-conditioned medium (N2B2 preconditioned by B104 neuroblastoma cells), 5 ng/ml FGF-2 and 0.5% FBS]. OPC cultures were amplified for 4 days and passaged once using papain (Young and Levison, 1997) prior to experiments. To obtain highly enriched cultures of late OPCs for experiments, early OPCs were replated at 2 × 10^4^ cells/cm^2^ on to poly-D-lysine-coated dishes in N2S with 0.5% FBS and 10 ng/ml FGF-2 for 48 h. These conditions produced cultures that contain 90–95% 04^+^/Ranscht^−^ late OPCs, 6–8% R24^+^/04^−^ early OPCs, 1–2% Ranscht^+^ immature oligodendrocytes, 2% GFAP^+^ astrocytes and 0.01% OX42^+^ microglia (Ness and Wood, 2002). Treatment medium was N2B2 medium without insulin, FGF-2 and FBS. Cells were treated for various time points with 500 μM glutamate and/or 20 ng/ml of IGF-1; media was replaced every 18 h.

### Immunocytochemistry

OPCs were plated on to round 12 mm cover glasses (Fisherbrand) coated with 0.1 mg/ml poly-L-ornithine in N2S media at a density of 2×10^4^ cells/cm^2^. Cells were differentiated to late OPCs and treated with glutamate or IGF-I as described above. Following treatments, cells were rinsed in PBS and fixed in 4% paraformaldehyde for 15 min at room temperature. OPCs were then rinsed three times in PBS and blocked for 30 min in 2% normal goat serum, 0.1% Triton X-100 in PBS at room temperature. Primary antibodies against Bax (1:50) or cofilin (1:50) were incubated in diluent (2% normal goat serum, 0.1% Triton X-100, 5 mg/ml BSA in PBS) overnight at 4°C. After three washes in PBS, primary antibodies were detected with fluorescein-conjugated goat-anti-mouse IgG (H+L) AF546 (Alexa Fluor 546) (red) and anti-rabbit IgG (H+L) AF488 (Alexa Fluor 488) (green) (1:2000) (Invitrogen) secondary antibodies for 45 min at room temperature. Incubation for 5 min with DAPI (1:1000) was used to identify nuclei. Coverslips were washed three times in PBS and mounted on slides with Fluoro-Gel (EMS).

### Immunoprecipitation (IP) and Western blotting

Late OPCs were treated with glutamate and/or IGF-I for 24 h. Cells were harvested in ice-cold PBS with PIC (protease inhibitor cocktail, 1:100; Sigma) and PMSF (1 μM; Sigma). For Bax(6A7) IP, cells were lysed in 1% CHAPS lysis buffer [10 mM Hepes, 150 mM NaCl, PIC (1:100), 20 μM PMSF, 5 μM NaF, 1 μM Na_3_VO_4_, 50 μM NaH_2_PO_4_]. For all other IPs, cells were lysed in 1% NP-40 lysis buffer [150 μM NaCl, 5 μM EDTA, PIC (1:100), 20 μM PMSF, 1 μM Na_3_VO_4_, 5 μM NaF]. After sonification, samples were quantified by Protein DC Assay (Bio-Rad Laboratories). For IPs 500–1000 μg of protein (or 2 mg for protein from white matter dissections) was incubated with the appropriate optimized amount of antibody overnight at 4°C on a rotating shaker according to the protocol from Pierce. The antigen–antibody complex was incubated with immobilized Protein A/G with gentle mixing for 2 h at 4°C on a rotating shaker. The complex was washed three times in 25 μM Tris/HCl (pH 7.5), 150 μM NaCl. For all Western blot analyses, 15–25 μg of protein was heated in electrophoresis loading buffer to 85°C for 5–10 min and separated on 12% mini-SDS polyacrylamide gels (Invitrogen). Proteins were then electrotransferred on to nitrocellulose membranes (Whatman). Membranes were blocked with appropriate primary and secondary antibodies (GAR-HRP or GAM-HRP conjugated IgG from Jackson ImmunoResearch) in 5% milk in TBS/0.1% Tween 20. Secondary antibody was detected using an enhanced chemiluminescence system (New England Nuclear). Images were digitized on an Ultra-lum Gel Imager (BioVision). Controls for IP reactions included isotope specific IgG (mouse, rabbit; Santa Cruz Biotechnology) and sample minus antibody incubation.

### Amnis ImageStream imaging flow cytometry

Late OPCs (1×10^6^) in suspension were prepared by harvesting cells using 0.05% Trypsin/EDTA (Cellgrow, Mediatech), followed by centrifugation at 1000 ***g*** for 5 min. The cell pellet was washed with 1×PBS, resuspended in 500 μl of 4% paraformaldehyde and fixed overnight at 4°C. Cells were again collected by centrifugation and the cell pellet permeabilized in buffer (0.1%Triton X-100 in PBS), washed with 2% FBS in PBS and incubated with primary antibody against Bax (1:50; Santa Cruz Biotechnology) and CoxIV (1:50; Cell Signaling Technology) for 30 min at 4°C. Cells were washed and incubated with secondary antibody (AF488-green, AF647-red, 1:2000) for 30 min. Cells were rinsed with 2% FBS and resuspended in 1% paraformaldehyde. Samples were acquired on the Amnis Imagestream 100 with the EDF element. Samples were then analyzed with IDEAS 4.0. Briefly, single cells were identified by graphing the area of the BF (brightfield) vs Aspect Ratio Intensity. Single cells were then analyzed for double-positive AF488 and AF647 by graphing Intensity AF488 vs AF647. Double-positive cells were finally analyzed for co-localization by the Bright Detail Similarity feature for the two stains. A value of 1.75 or greater denoted co-localized events.

### Subcellular fractionation

Mitochondrial/cytoplasmic fractionation was performed using the Mitochondria Isolation Kit for Cultured Cells (Pierce) with minor modifications. Late OPCs (2 × 10^7^) were harvested by centrifuging at 1000 ***g*** for 2 min. Following separation, the mitochondrial and cytoplasmic fractions were lysed in 2% CHAPS in Tris-buffered saline (25 mM Tris, 0.15 M NaCl, pH 7.2) and protein concentration was determined by the DC protein Assay (Bio-Rad Laboratories).

### Mass spectrometry and protein identification

OPCs were differentiated to the late OPC stage and then treated with glutamate or IGF-I as for prior experiments for 28 h and lysed in 1% CHAPS lysis buffer. Immunoprecipitation was performed for total Bax (N20) and then proteins were separated by SDS/PAGE. After electrophoresis, the gel was fixed and subjected to SYPRO Ruby staining, and gel bands were excised, diced into 1 mm^3^ pieces and washed with 30% ACN (acetonitrile) in 50 mM ammonium bicarbonate followed by reduction with DTT and alkylation by iodoacetamide. Digestion was initiated by adding trypsin and incubating at 37°C overnight. The resulting peptides were extracted with 30 μl of 1% TFA (trifluoracetic acid) followed by C_18_ Ziptip (Millipore) desalting according to the manufacturer's protocol. The peptides were dried in a Speedvac, and re-suspended in 10 μl of solvent A [2% ACN, 0.1% FA (formic acid)] for ESI-LC-MS/MS analysis on a API-US QTOF tandem MS system equipped with a nano-ESI source (Waters Corporation) as described previously (Wu et al., 2011). Protein identification was performed by searching the MS/MS spectra against rat protein sequences in the NCBInr database using a Mascot search engine (version 2.4.1). Precursor mass tolerance was set at 100 p.p.m. and fragment mass tolerance was set at 0.6 Da. Oxidized methionine and carbamidomethyl-labelled cysteine were set as variable modifications as the search parameters. Mascot search results were further exported to Scaffold software (version 3.6.5) for visualization and validation. Peptide identifications were accepted if they could be established at greater than 95.0% probability by the Peptide Prophet algorithm (Keller et al., 2002). Protein identifications were accepted if they could be established at greater than 95.0% probability and contained at least 1 identified peptides. Protein probabilities were assigned by the Protein Prophet algorithm (Nesvizhskii et al., 2003). Proteins that contained similar peptides and could not be differentiated based on MS/MS analysis alone were grouped to satisfy the principles of parsimony.

### Perinatal H–I and white matter dissections

Timed pregnant Wistar rats were obtained from Charles River Laboratories. After normal delivery litter size was adjusted to 12 pups per litter. Two different groups of animals were used: sham-operated controls and experimental H–I animals. Cerebral H–I was produced in male and female 6-day-old rats (day of birth=P0) by a permanent unilateral common carotid ligation followed by hypoxia (variation of the Vannucci Model) (Rice et al., 1981; Romanko et al., 2007). Briefly, pups were lightly anesthetized with halothane (4% induction and 1.5% maintenance). Once fully anesthetized, a midline neck incision was made and the right CCA (common carotid artery) was identified. The CCA was separated from the vagus nerve and then cauterized at two distinct locations with a bipolar cauterizer at power setting of 10 (Codman & Shurtleff, model number 80-1140). Animals were returned to the dam for 1.5 h. The pups were pre-warmed in jars for 20 min submerged in a 37°C water bath and then exposed to 75 min of hypoxia (8% O_2_/92% N_2_). After hypoxia the pups were returned to their dam for recovery periods of 24 h, at which time they were anesthetized and killed by intracardiac perfusion. Sham-operated animals were anesthetized and the surgery was performed without ligation of the right CCA followed by exposure to hypoxia.

All brain tissue samples (*n*=6 per experiment for H–I and sham) were dissected fresh and pooled [IL (ipsilateral) and CL (contralateral) were pooled separately for H–I brains] and homogenized on ice in 500 μl of homogenization buffer with a Tissue-Tearor (rotor/stator type) homogenizer (BioSpec Products). Homogenization buffer contained 1% NP-40 lysis buffer [150 μM NaCl, 5 μM EDTA, PIC (1:100), 20 μM PMSF, 1 μM Na_3_VO_4_, 5 μM NaF] and 1% CHAPS lysis buffer [10 mM Hepes, 150 mM NaCl, PIC (1:100), 20 μM PMSF, 5 μM NaF, 1 μM Na_3_VO_4_, 50 μM NaH_2_PO_4_]. Samples were used for IPs or Western blotting as described above.

## RESULTS

### Time course of Bax activation and mitochondrial translocation in late OPCs after exposure to excitotoxic levels of glutamate *in vitro*

Our *in vitro* model for glutamate-mediated excitotoxicity of OPCs utilizes media where the IGF-1R (IGF type 1 receptor) is not stimulated (by eliminating micromolar levels of insulin present in most medium formulations) and it does not contain cyclothiazide, the glutamate-densensitizing blocker (Ness and Wood, 2002; Ness et al., 2004; Wood et al., 2007). Under these conditions, apoptosis occurs between 24 and 36 h following exposure to glutamate (Ness et al., 2004). This paradigm also has allowed us to study glutamate excitotoxicity as well as the effects of IGF-I in preventing glutamate-induced apoptosis. Thus, in our previously published and the current studies, we use low-insulin media supplemented with IGF-I for healthy OPCs, and the same media plus glutamate either in the presence or absence of IGF-I (Ness et al., 2004; Wood et al., 2007). Here, we use this culture paradigm to further define molecules involved in the apoptotic cascade in OPCs and to determine how a trophic factor such as IGF-I interferes with the cascade.

**Figure 1:**
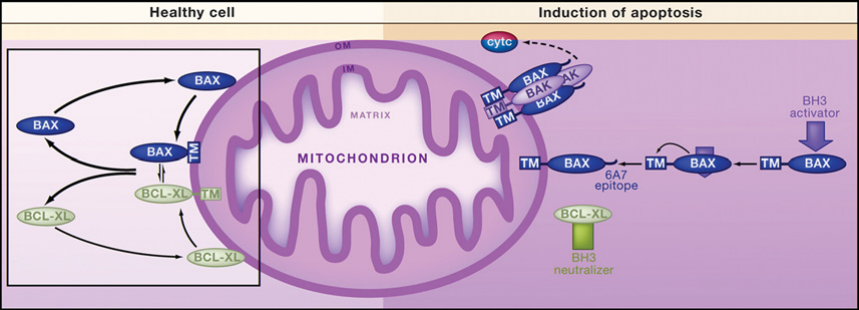
Bax movements in healthy cells and during apoptosis In healthy cells, inactive Bax continuously cycles between mitochondria and the cytosol. Bax retrotranslocation requires interaction with an anti-apoptotic protein (Bcl-xL, Bcl-2, or Mcl1). Together, these two proteins leave the mitochondrial outer membrane (OM). Once in the cytosol, the complex immediately dissociates. The retrotranslocation process is stimulated by the anti-apoptotic proteins Bcl-xL, Bcl-2, or Mcl1 and is inhibited by vMIA, ABT-737, and BH3-only proteins. Upon induction of apoptosis, Bax is directly stimulated by activating BH3-only proteins (e.g., Bid, Bim, or Puma; blue arrow) to expose its C-terminal domain and insert in the mitochondrial OM. During this process, Bax exposes a novel N-terminal epitope (6A7), triggering the formation of foci and release of cytochrome *c*. Neutralizing BH3-only proteins (or small molecule inhibitors; green rectangle) can indirectly activate Bax by binding and inactivating antiapoptotic proteins. Consequently, Bax accumulates on the mitochondrial OM, where it acquires its active conformation. Figure and legend reprinted from, Cell 145(1), M.E. Soriano and L. Scorrano, Traveling Bax and Forth from Mitochondria to Control Apoptosis, Pages 15–17, (2011), with permission from Elsevier.

**Figure 2:**
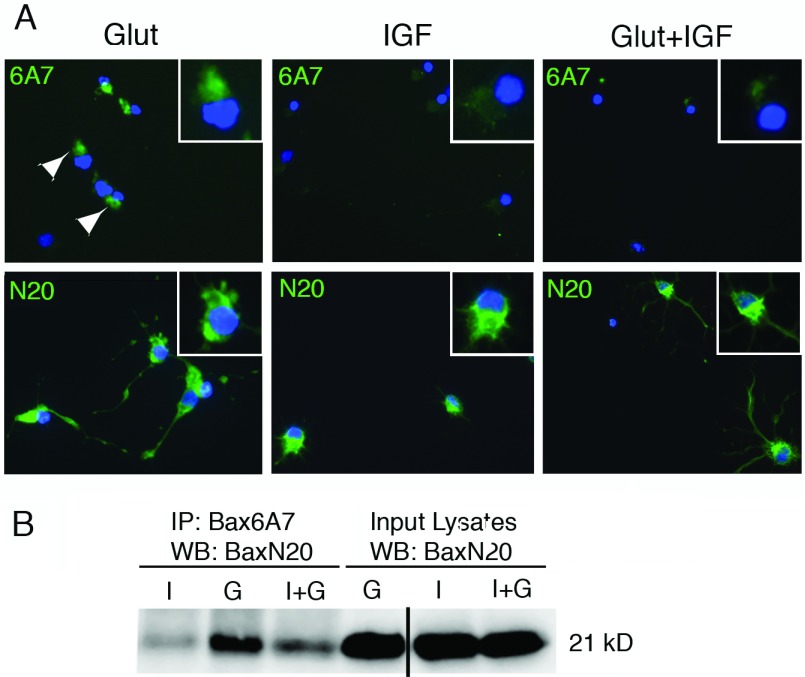
Detection of active Bax in OPCs exposed to glutamate Late OPCs were treated with glutamate (500 μM)±IGF-I (20 ng/ml) for 24 h. (**A**) Immunostaining for conformation-specific Bax (6A7; upper panels) or total Bax (N20; lower panels). Bax was detected using an AF488-conjugated secondary antibody (green); DAPI-stained nuclei are shown as blue. (**B**) Active Bax was detected by immunoprecipitation from glutamate-treated OPCs using conformation-specific Bax antibody (6A7) followed by Western blotting with total Bax N20 antibody (21 kDa). I, IGF-I; G, glutamate; I+G, IGF-I plus glutamate. Data are representative of two independent experiments at 24 h. Similar results were obtained at 18 h. Line in gel in (**B**) indicates that a blank lane was removed from the gel for publication purposes.

In healthy cells, endogenous Bax is a monomeric protein in an inactive conformation (Vogel et al., 2012). Following a death stimulus, Bax is activated, translocates to the mitochondria and oligomerizes (Gross et al., 1998; Edlich et al., 2011; Soriano and Scorrano, 2011; Walensky and Gavathiotis, 2011) (Figure 1). In our prior studies we showed Bax associated with mitochondria in OPCs exposed to glutamate, but not in the presence of IGF-I (Ness et al., 2004); however, we did not assess the activation state of Bax. After exposure to apoptotic stimuli, an N-terminal epitope of Bax is exposed and involved in Bax membrane insertion (Sharpe et al., 2004). To determine whether Bax undergoes conformational changes in OPCs during excitotoxicity, we determined whether conformation-specific Bax was present in OPCs prior to apoptotic death. We detected activated Bax in OPCs exposed to glutamate at 24 h using the conformation specific antibody 6A7 (Figure 2A, upper panels; Glut, arrowheads) but not in cells cultured in IGF-I either in the absence (IGF-I) or presence (Glut+IGF) of glutamate. Total Bax was detected in cells cultured under all conditions (Figure 2A, lower panels).

To confirm the presence of active Bax in OPCs exposed to glutamate, we immunoprecipitated active Bax using the Bax conformation-specific antibody followed by Western blotting to detect total Bax protein. Consistent with the immunofluorescence results, there was more active Bax present after 24 h in OPCs exposed to glutamate and less active Bax present in OPCs stimulated with glutamate and IGF-I (Figure 2B). Similar results were obtained as early as 18 h after exposure to glutamate (data not shown).

**Figure 3:**
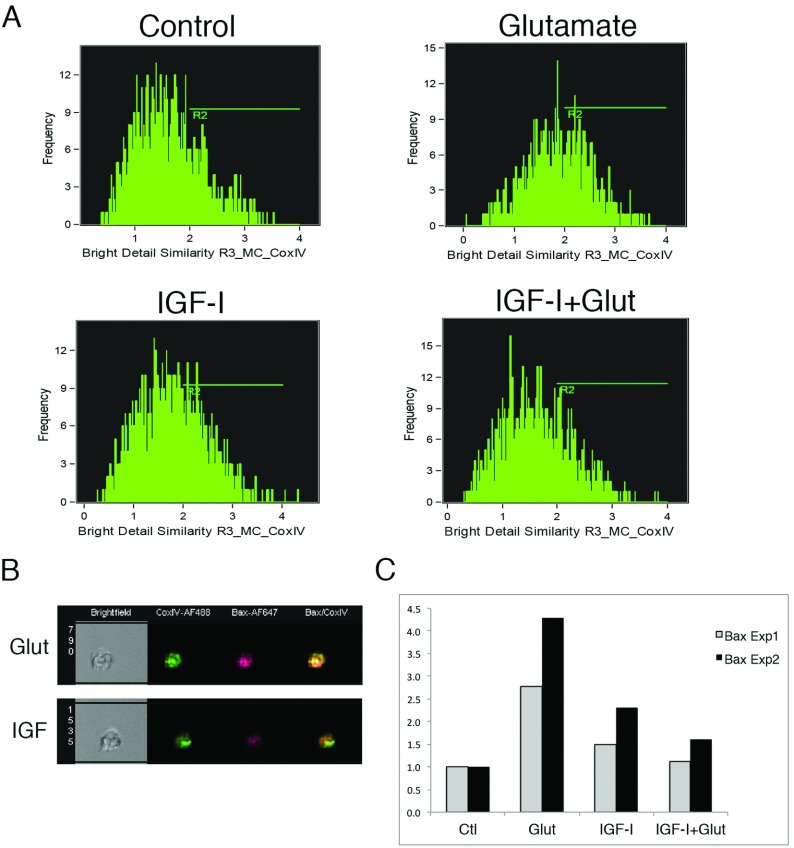
Bax associates with mitochondria by 18 h in OPCs exposed to glutamate Late OPCs were treated with glutamate (500 μM)±IGF-I (20 ng/ml) for 18 h. (**A** and **B**) Quantification of cells with Bax-CoxIV co-localization using the Amnis ImageStream System. (**A**) Shift of peak to the right vs controls indicates increased co-localization in glutamate-treated OPCs. (**B**) Images show examples of Bax (N20)-CoxIV co-localized after glutamate stimulation (Glut) and reduced co-localization upon addition of IGF-I (IGF-I). (**C**) Histogram showing fold increase of Bax association with CoxIV in two independent experiments. For each experiment, an average of 1500 individual cells were evaluated for each condition. An increase in Bax association with CoxIV was also observed at 24 h.

In our prior studies, we demonstrated Bax co-localization with mitochondria using immunofluorescence microscopy (Ness et al., 2004). To quantify Bax translocation to mitochondria in OPCs exposed to excitotoxic levels of glutamate, we used using Amnis Imagestream 100 multispectral imaging flow cytometry (Figure 3), a quantitative method performed on thousands of individual cells with each cell considered an independent event and used to determine a similarity score indicative of co-localization. OPCs treated with glutamate and/or IGF-I were stained for Bax and CoxIV, a mitochondrial outer membrane marker. Co-localization was determined by calculating the similarity of two channels and similarity above a threshold indicated by R2 (Figures 3A and 3B). In glutamate-treated OPCs, there was an approximately 3**–**4-fold increase in the percentage of cells where Bax and CoxIV co-localized at 18 h versus IGF-I- or IGF-I+glutamate-treated OPCs (Figure 3C). Similar results were obtained at 24 h, however, since CoxIV levels begin to decrease once mitochondria are disrupted, these analyses are shown at the 18 h timepoint, which precedes cytochrome *c* release, caspase 9 activation and the appearance of apoptotic nuclei (Ness et al., 2004).

**Figure 4:**
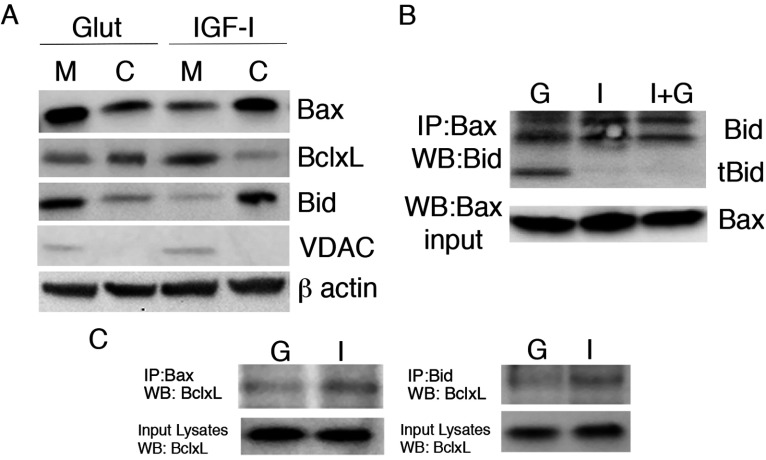
Bcl-2 family regulation in OPCs during glutamate excitotoxicity (**A**) Mitochondrial (M) and cytoplasmic (C) fractions were analyzed for Bax, Bid and Bcl-xL in OPCs treated with glutamate (500 μM) or IGF-I (20 ng/ml) for 24 h. The mitochondrial protein VDAC was used to confirm fractionation. (**B** and **C**) Isolated protein was used to immunoprecipitate total Bax (N20) or Bid from OPCs treated for 24 h with glutamate (G), IGF-I (I) or IGF-I+glutamate (I+G). Bid and tBid were detected by Western immunoblotting (**B**). Bcl-xL was seen associated with Bax and Bid in IGF-I-treated OPCs and reduced in glutamate-treated OPCs (**C**). Subcellular fractionation data are representative of two independent experiments. IPs are representative of two independent experiments at 24 h; similar results were obtained at 18 h for detection of Bcl-xL associated with Bax or Bid.

Using subcellular fractionation, we further investigated the accumulation of pro-apoptotic and anti-apoptotic proteins in mitochondria and cytoplasmic fractions after 24 h of glutamate or IGF-I treatment (Figure 4). We observed higher levels of Bax protein in the mitochondrial fraction compared with the cytoplasmic fraction in glutamate-treated cells (Figure 4A) consistent with our prior immunodetection of Bax translocation to the mitochondria 24 h after exposure to glutamate (Ness et al., 2004). In IGF-I-treated cells, Bax levels were higher in the cytoplasmic fraction (Figure 4A). Bcl-xL, a member of the pro-survival Bcl-family that is abundant in OPCs (Itoh et al., 2003), partitioned preferentially to the mitochondrial fraction in IGF-I-treated OPCs whereas it translocated to the cytoplasmic fraction after glutamate treatment (Figure 4A). This pattern was the reverse of what was observed for Bax in glutamate vs IGF-I conditions. These data on Bax activation and translocation demonstrate that OPC exposure to glutamate results in Bax activation and mitochondrial translocation, all of which can be inhibited by IGF-I. Moreover, the enrichment of Bcl-xL in the mitochondrial fraction in IGF-I/survival conditions supports the hypothesis that Bcl-xL antagonizes Bax-mediated apoptosis in OPCs. However, based on their inverse localization, Bcl-xL may antagonize Bax in OPCs, at least in part, through indirect mechanisms as reported in other cell types (Willis and Adams, 2005; Willis et al., 2007; Billen et al., 2008; Lovell et al., 2008).

### Pro-apoptotic BH3-domain partners of Bax identified in late OPCs

One of the best-studied Bax activators is the BH3-only protein Bid, which binds pro-apoptotic Bax and Bak as well as anti-apoptotic Bcl-2 and Bcl-xL (Willis and Adams, 2005; Willis et al., 2007). Bid is cleaved by caspase 8 or caspase 3 (Bossy-Wetzel and Green, 1999; Slee et al., 1999; Yin, 2006) and the resulting tBid (truncated Bid) translocates from the cytoplasm to mitochondrial membranes. Indeed, Bax oligomerization and insertion into the outer mitochondrial membrane can be triggered by tBid (Lovell et al., 2008). Real-time PCR analyses have shown that Bid is expressed in oligodendrocytes at all stages of development (Itoh et al., 2003). Our analyses of Bid revealed that Bid partitioned to the cytoplasm in healthy cells (IGF-I conditions) but that it translocated to the mitochondrial fraction after glutamate-treatment (Figure 3A). Furthermore, tBid co-immunoprecipitated with Bax after glutamate-treatment, whereas it was undetectable in cells treated with either IGF-I or IGF-I plus glutamate (Figure 4B), suggesting that IGF-I blocks glutamate-mediated apoptosis upstream of Bid cleavage and association with Bax.

We further analyzed the association of the anti-apoptotic protein Bcl-xL with Bax and Bid in OPCs by co-immunoprecipitation. OPCs stimulated with glutamate had reduced Bax–Bcl-xL association as well as reduced association of Bid–Bcl-xL vs cells treated with IGF-I (Figure 4C). These data support a model where tBid enhances Bax-mediated apoptosis in OPCs and Bcl-xL inhibits Bax activation under survival conditions through both direct and indirect mechanisms.

### Identification of cofilin as a Bax-binding partner in OPCs

**Figure 5:**
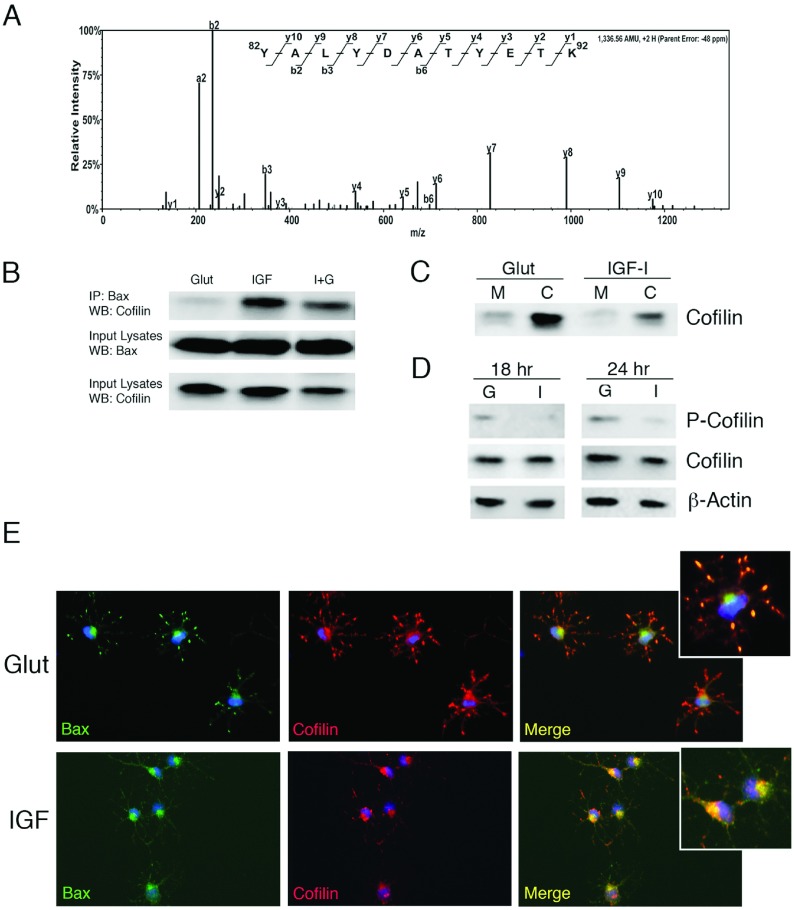
Identification of cofilin as a Bax-binding partner in IGF-I-treated OPCs (**A**) Bax was immunoprecipitated from OPCs treated for 24 h with IGF-I. The protein band corresponding to ~20 kDa was excised from the gel and subjected to ESI–LC–MS/MS analysis. Scaffold-generated MS/MS spectrum of a doubly charged ion corresponds to Y^82^ALYDATYETK^92^ peptide from cofilin-2 (NCBI Reference Sequence NP_001102452.2). The observed y- and b-ion series confirmed the peptide sequence. (**B**) Isolated protein from OPCs treated for 24 h with glutamate or IGF-I was used to immunoprecipitate total Bax (N20). Cofilin was detected by Western immunoblotting in IGF-I-treated OPCs. Association of cofilin with Bax was barely detectable in OPCs treated with glutamate at 24 h. Data are representative of two experiments; similar results were obtained at 18 h. (**C**) Analysis of cofilin in mitochondrial (M) and cytoplasmic (C) fractions 24 h following exposure of OPCs to glutamate or IGF-I. (**D**) Detection of p-cofilin (Ser^9^) in OPCs treated with glutamate (G) but not in OPCs treated with IGF-I (I) at 18 h and 24 h. (**E**) Immunodetection of Bax (N20; AF488-green) and cofilin (AF546-red) in OPCs treated with either glutamate (Glut) or IGF-I (IGF). DAPI (blue) was used to detect nuclei. Data are representative of at least two independent experiments.

To identify novel binding partners of Bax in both glutamate and IGF-I-treated late OPCs, we immunoprecipitated Bax and utilized a LC-MS/MS proteomic approach to identify associated proteins. One protein we identified as a Bax interacting protein in IGF-I-treated, but not glutamate-treated, OPCs was cofilin, a member of the ADF (actin depolymerizing factor)/cofilin family of actin-binding proteins, that prevents reassembly of actin filaments (Bernstein and Bamburg, 2010) (Figure 5A). Across several independent experiments, we confirmed that cofilin co-immunoprecipitated with Bax in IGF-I-treated, but not in glutamate-treated, OPCs (Figure 5B). Prior studies have demonstrated that cofilin associates with mitochondria during apoptosis (Chua et al., 2003; Klamt et al., 2009). Specifically, cofilin was shown to translocate to the mitochondria in several human cell lines prior to cytochrome *c* release following induction of apoptosis (Chua et al., 2003). Moreover, perturbations that reduced cofilin prevented apoptosis; cofilin localization to mitochondria promoted apoptosis in these studies (Chua et al., 2003). Therefore, we investigated whether cofilin shifted localization from the cytoplasm to the mitochondria, similar to Bax in OPCs exposed to glutamate. These studies established that cofilin preferentially partitioned to the cytoplasm in both IGF-I- and glutamate-treated cells (Figure 5C). Since others have shown that phosphorylation of cofilin at Ser^9^ (p-cofilin) is associated with its inactivation (Klamt et al., 2009; Posadas et al., 2012), we determined whether the phosphorylation status of cofilin changed in glutamate-stimulated OPCs. These studies revealed that p-cofilin increased in glutamate-treated OPCs vs IGF-I-treated OPCs at both 18 h and 24 h (Figure 5D), suggesting it is in an active state in the presence of IGF-I but is inactivated during excitotoxic death. Immunostaining for cofilin and Bax in OPCs revealed that cofilin co-localized with Bax in the cell body in the IGF-I condition, but was dispersed into cell processes and showed less co-localization with Bax after glutamate stimulation (Figure 5E). Taken together, these data are inconsistent with the ascribed function of active, dephosphorylated cofilin in Bax translocation and mitochondrial dysfunction. Rather, our data are more consistent with the hypothesis that cofilin in late OPCs regulates actin reorganization during process outgrowth, which may include regulating mitochondria and/or Bax indirectly.

### Bax regulation in white matter following hypoxia–ischemia in the immature brain

To determine mechanisms of apoptosis and Bax regulation in OPCs *in vivo*, we used the Vannucci neonatal rat model of hypoxic–ischemic injury. In prior studies, we documented increased apoptosis of OPCs in white matter of the corpus callosum within 24 h after inducing H–I in 6-day-old neonatal rat pups (Ness et al., 2001). We also demonstrated that IGF-I rescued these cells from cell death in a separate set of studies (Wood et al., 2007). Extending these earlier studies, we micro-dissected the subcortical white matter at 24 h of recovery from H–I and evaluated Bid and Bcl-xL association with Bax. Since the Vannucci H–I model produces unilateral hemispheric damage, we examined the ipsilateral hemisphere (IL-damaged, right) and contralateral hemisphere (CL-spared, left), in addition to sham operated brains (controls).

**Figure 6:**
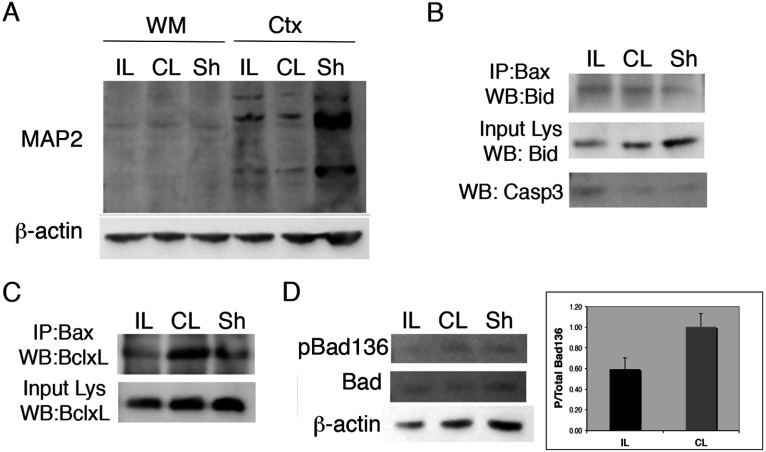
Bcl-2 family regulation in immature white matter after H–I H–I was produced in P6 Wistar rat pups by cauterizing the common carotid artery followed by systemic hypoxia for 75 min. Subcortical white matter (WM) was dissected at 24 h recovery from ipsilateral (IL), contralateral (CL) and Sham (Sh) operated brains and used for protein isolation (*n*=6 pooled samples). (**A**) Western blot of MAP-2 expression in cortex vs WM dissected regions. (**B**) Active caspase 3 increased in IL white matter (bottom panel). (**B-C**) Isolated protein was used to immunoprecipitate total Bax (N20). Bax-associated Bid (**B**) and Bcl-xL (**C**) were detected by Western immunoblotting. The data for the Bax–BclxL association are representative of three independent experiments and the data for the Bax–Bid association are from two independent experiments. (**D**) Detection of pBad(Ser^136^) and total Bad by Western immunoblotting of protein from IL, CL and Sh white matter. Histogram shows ratio of p-Bad/total Bad in IL vs CL white matter. Values represent averages±S.D. from three independent experiments; *P* =  0.08).

To validate the purity of our white matter dissections, we analyzed expression of MAP2, a dendrite-restricted protein that is enriched in gray matter and absent from white matter (Bradke and Dotti, 2000; Iwata et al., 2005) (Figure 6A). We also evaluated levels of cleaved caspase 3 in the IL white matter to confirm the increase in apoptotic death in the H–I white matter (Figure 6B). These analyses revealed low levels of MAP-2 in the micro-dissected white matter and an increase in active caspase 3 in the IL vs CL and sham-operated white matter. Bax immunoprecipitation revealed increased association with full length Bid in both IL and CL white matter; however, tBid was undetectable in either sample (Figure 6B). In contrast, Bax association with Bcl-xL was readily apparent in the CL white matter and reduced in the IL white matter (Figure 6C).

Based on the absence of any detectable tBid associated with Bax in the damaged white matter, we expanded our studies to include an analysis of the pro-apoptotic, BH3-only protein Bad. Bad is a positive regulator of cell death and like other BH3-only proteins, selectively displaces Bax from heterodimerizing with Bcl-2 or Bcl-xL (Willis and Adams, 2005; Willis et al., 2007). In the absence of survival stimuli, endogenous Bad is dephosphorylated and localized in the mitochondrial outer membrane; in the presence of survival factors, Bad is phosphorylated at serine sites 136, 112, and 155 (Datta et al., 2000). We detected decreased pBad (Ser^136^) in the IL white matter at 24 h after H–I whereas there was no difference between CL and sham-operated white matter; no difference in total Bad was detected between conditions (Figure 6D). These data are consistent with a role for Bad in mediating apoptosis in the immature white matter following H–I.

## DISCUSSION

In OPCs, the pro-apoptotic Bcl2 protein Bax is a major mediator of apoptotic death due to excitotoxicity (Dong et al., 2003; Ness et al., 2004; Pang et al., 2007; Sanchez-Gomez et al., 2011). Despite numerous studies on Bax in a variety of cell types, there is still considerable debate about how Bax is activated, and there are no data to address mechanisms of Bax activation in OPCs. Here, we examined Bax regulation in glutamate excitotoxicity in late OPCs *in vitro* and following H–I in immature white matter. Our studies suggest potential roles for the BH3-only proteins Bid or Bad in activating Bax depending on the model of OPC death. However, excitotoxicity *in vitro* as well as white matter damage *in vivo* both correlate with loss of Bax association with Bcl-xL. Moreover, we identified cofilin as a Bax-associated partner in healthy OPCs and found that this association is disrupted in cells undergoing excitotoxic death.

### Excitotoxicity in OPCs involves tBid–Bax association and loss of Bcl-xL–Bax

**Figure 7:**
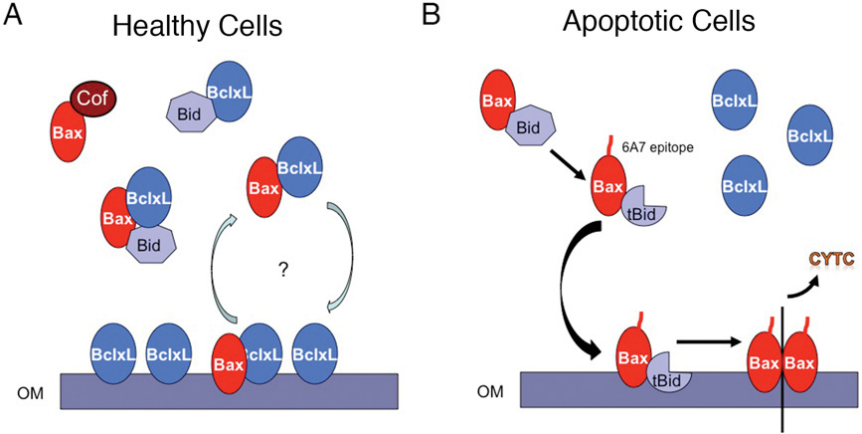
Model of Bax regulation in OPCs Schematic diagrams showing Bax binding partners, their associations and their locations in OPCs under survival conditions (**A**) or under apoptotic conditions following exposure to excess glutamate (**B**). Models are based predominantly on data obtained from the *in vitro* studies reported in this paper. Bax shuttling from mitochondria to cytoplasm by Bcl-xL is supported by published models (see Figure 1).

Bax is mainly present in the cytosol of healthy cells, but after apoptotic stimuli it can shuttle from the cytoplasm to ER (endoplasmic reticulum), mitochondria and nucleus (Gill et al., 2008; Ghibelli and Diederich, 2010) (Figure 1). Our studies demonstrate that excitotoxic levels of glutamate result in a Bax conformational change and increased association of Bax with mitochondria in OPCs. Bax oligomerization and insertion into the outer mitochondrial membrane can be triggered by tBid. Our studies demonstrate that both Bax and Bid translocate from the cytoplasm to mitochondria during the early stages of excitotoxic cell death and that tBid also associates with Bax in OPCs exposed to damaging concentrations of glutamate (Figure 7B). Recent studies on Bax–Bid function in cell-free systems demonstrate that tBid and Bax complex formation requires membrane association which leads to the insertion of Bax into the membrane and formation of membrane pores (Lovell et al., 2008). Taken together, our current and past data support the hypothesis that tBid–Bax binding contributes to mitochondrial permeabilization, cytochrome *c* release and apoptosis in OPCs during excitotoxicity. These data support a direct model of Bax activation where a BH3-only protein directly activates Bax (Chipuk and Green, 2008). Based on our prior data showing active caspase 3 during glutamate-mediated apoptosis of OPCs (Ness et al., 2004) and on a proposed model for Bid function in apoptosis (Slee et al., 2000), we hypothesize that Bid is cleaved downstream of caspase 3 to promote a caspase 3/tBid-dependent amplification loop. The initial trigger for Bid cleavage is unknown, however, likely candidates are calpains. Other studies have shown that calpains are responsible for the initial cleavage of Bid (Yin, 2006), and that calpains have demonstrated functions in Bax-mediated death in OPCs downstream of calcium influx through AMPA/kainate receptor activation (Sanchez-Gomez et al., 2011).

Bcl-xL, an anti-apoptotic member of the Bcl-2 family, is highly expressed in OPCs (Itoh et al., 2003; Ness et al., 2004). Two models have been proposed to explain how Bcl-xL inhibits Bax: (i) Bcl-xL binds to BH3-only proteins such as Bid, preventing Bax activation, and (ii) Bcl-xL directly binds to activated Bax to inhibit it from forming membrane pores (Chipuk and Green, 2008). We observed no complex formation between Bcl-xL and the active form of Bax (data not shown). In healthy OPCs Bcl-xL preferentially attached to the mitochondria whereupon it shifted to the cytoplasm during excitotoxicity. Recently, it was proposed that inactive Bax preferentially associates with the mitochondrial outer membrane and that anti-apoptotic proteins are required to constitutively retrotranslocate Bax into the cytosol (Edlich et al., 2011) (see Figure 1). Bcl-xL may regulate Bax in this fashion in OPCs, but the association must be transient since our cell fractionation studies indicate an inverse relationship between Bcl-xL and both Bax and Bid in healthy OPCs, such that Bax and Bid are predominantly cytoplasmic and Bcl-xL is predominantly mitochondrial (Figure 7A). Our data also support an alternative model where Bcl-xL inhibits Bax by competing with it for binding to organelle membranes (Billen et al., 2008).

### Cofilin binds Bax in healthy OPCs and binding is disrupted during excitotoxic death

Our identification of cofilin as a binding partner of Bax in OPCs is both novel and intriguing based on prior literature. Cofilin is a member of the cofilin/ADF family that regulates actin dynamics by increasing the rate of actin depolymerization. Cofilin sequesters G-actin, severs F-actin, and is abundant in growth cones and presynaptic terminals (Fox et al., 2006; Bernstein and Bamburg, 2010; Marsick et al., 2010; Mizuno, 2013). In mammalian cell lines treated with staurosporine, cofilin was identified by a proteomic approach as a factor that translocated from the cytosol into the mitochondria before cytochrome *c* release (Chua et al., 2003). In a recent study that evaluated the mechanisms of NMDA-induced cortical neuronal cell death, cofilin was found to be dephosphorylated and activated resulting in its translocation with Bax to the mitochondria (Posadas et al., 2012). However, our data demonstrate that in OPCs cofilin binds to Bax only in IGF-treated OPCs and is predominantly cytoplasmic in OPCs in both IGF-I- and glutamate-treated conditions. Moreover, the presence of phosphorylated cofilin in OPCs undergoing excitotoxic death is inconsistent with active, dephosphorylated cofilin mediating Bax translocation and mitochondrial dysfunction. However, cofilin also has known functions in actin reorganization, which may include regulating mitochondria and/or Bax indirectly in OPCs. For example, it has been shown that mitochondria associate with actin prior to Bax translocation (Tang et al., 2006), thus cofilin phosphorylation and inactivation may function to stabilize the actin cytoskeleton to promote this association.

### Regulation of Bax after H–I in white matter

White matter damage after H–I in the immature brain is believed to result from vulnerability of the immature oligodendrocyte (the late OPC) to factors elevated during ischemic damage, such as oxygen free radicals and glutamate (Volpe, 2001; Back et al., 2007; Segovia et al., 2008; Buser et al., 2010, 2012). Similar to the glutamate-mediated pathway of Bax activation we observed in OPCs *in vitro*, Bax–Bcl-xL association was disrupted after neonatal H–I in white matter. Although neonatal white matter is not exclusively comprised of OPCs, these data support a model where Bax is bound to Bcl-xL in healthy OPCs and the release of Bax from Bcl-xL is a component of its activation. However, in contrast with the *in vitro* studies, we failed to detect tBid associated with Bax in the H–I white matter. This could be due either to low levels of Bax–tBid in the OPCs that were undetectable in extracts of total white matter or to a different BH3-only protein responsible for Bax activation in OPCs after H–I. In support of the latter possibility, we found reduced p-Bad (Ser^136^) in the damaged white matter. Bad functions to bind and inactivate anti-apoptotic proteins including Bcl-xL to promote the formation of the mitochondrial permeability transition pore (Chipuk and Green, 2008; Roy et al., 2009). These data suggest that Bad may be involved in Bax activation in OPCs after H–I. However, we were unable to convincingly detect changes in p-Bad in glutamate- vs IGF-I-treated OPCs *in vitro*, suggesting either that the two paradigms differ in which BH3-only proteins are involved in Bax activation and/or that the reduction in p-Bad in damaged white matter occurs in cells other than oligodendroglia.

In conclusion we have begun to define interactions of Bcl-2 family members with Bax in OPC apoptosis induced by glutamate excitotoxicity *in vitro* and in white matter damage after H–I *in vivo*. Bax activation and dissociation of Bax with Bcl-xL are common to both paradigms whereas the involvement of specific BH3-only proteins may vary contextually. Understanding the pathways and molecules involved in OPC cell death will provide insights towards developing new therapeutic interventions to promote survival of this lineage in brain and spinal cord injuries and in neurodegenerative diseases.
